# Genetic and molecular analysis of the anthocyanin pigmentation pathway in *Epimedium*

**DOI:** 10.3389/fpls.2023.1133616

**Published:** 2023-03-27

**Authors:** Yaolei Mi, Ruikun He, Huihua Wan, Xiangxiao Meng, Di Liu, Wenjun Huang, Yanjun Zhang, Zubaida Yousaf, Hongwen Huang, Shilin Chen, Ying Wang, Wei Sun

**Affiliations:** ^1^ Laboratory of Beijing for Identification and Safety Evaluation of Chinese Medicine, Institute of Chinese Materia Medica, China Academy of Chinese Medical Sciences, Beijing, China; ^2^ By-Health Institute of Nutrition and health. By-health Co., Ltd., Guangzhou, Guangdong, China; ^3^ College of Pharmacy, Hubei University of Chinese Medicine, Wuhan, Hubei, China; ^4^ Key Laboratory of Plant Germplasm Enhancement and Specialty Agriculture, Wuhan Botanical Garden, Chinese Academy of Sciences, Wuhan, Hubei, China; ^5^ Department of Botany, Lahore College for Women University, Lahore, Pakistan; ^6^ Lushan Botanical Garden, Chinese Academy of Sciences, Jiujiang, Jiangxi, China; ^7^ Institute of Herbgenomics, Chengdu University of Traditional Chinese Medicine, Chengdu, Sichuan, China; ^8^ South China Botanical Garden, Chinese Academy of Sciences, Guangzhou, Guangdong, China

**Keywords:** gene expression, *Epimedium*, anthocyanin, spur, sepal

## Abstract

**Introduction:**

Flower color is an ideal trait for studying the molecular basis for phenotypic variations in natural populations of species. *Epimedium* (Berberidaceae) species exhibit a wide range of flower colors resulting from the varied accumulation of anthocyanins and other pigments in their spur-like petals and petaloid sepals.

**Methods:**

In this work, the anthocyanidins of eight different *Epimedium* species with different floral pigmentation phenotypes were analyzed using HPLC. Twelve genes involved in anthocyanin biosynthesis were cloned and sequenced, and their expression was quantified.

**Results:**

The expression levels of the catalytic enzyme genes DFR and ANS were significantly decreased in four species showing loss of floral pigmentation. Complementation of EsF3’H and EsDFR in corresponding *Arabidopsis* mutants together with overexpression of EsF3’5’H in wild type *Arabidopsis* analysis revealed that these genes were functional at the protein level, based on the accumulation of anthocyanin pigments.

**Discussion:**

These results strongly suggest that transcriptional regulatory changes determine the loss of anthocyanins to be convergent in the floral tissue of *Epimedium* species.

## Introduction

**Figure 1:**
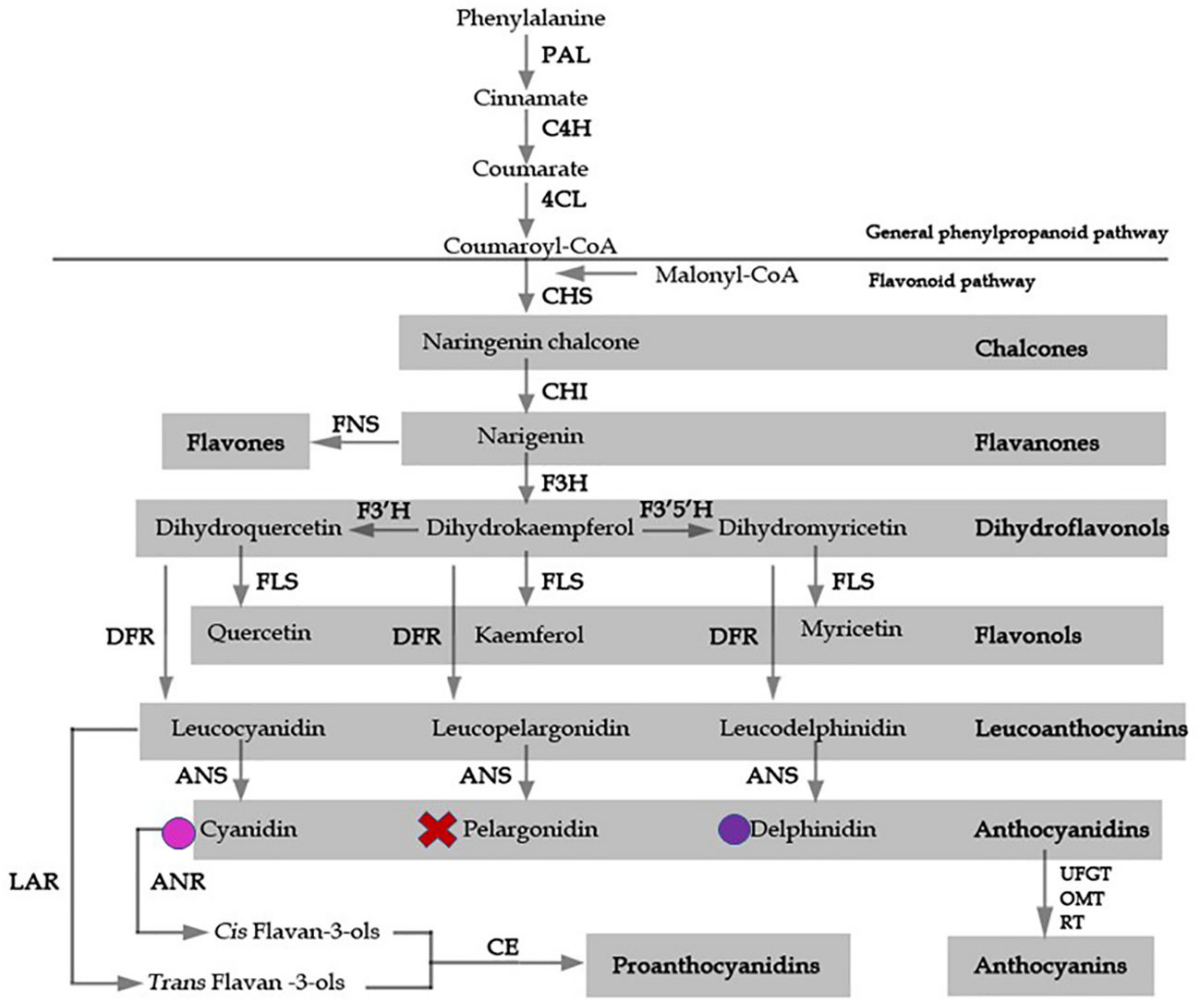
A model for flavonoid biosynthesis in *Epimedium* flowers based on classic investigation. Pathway enzymes are listed as an abbreviation beside arrows, and include 4CL, 4-coumarate: coenzyme A ligase; ANS, anthocyanidin synthase; C4H, cinnamate-4-hydroxylase; CHS, chalcone synthase, CHI, chalcone isomerase; DFR, dihydroflavonol 4-reductase; F3H, flavavone 3-hydroxylase; F3’H, flavonoid 3’-hydroxylase; F3’5’H, flavonoid 3’5’ hydroxylase; FLS, flavonol synthase; FNS, flavone synthase; LAR, leucoanthocyanidin reductase; UFGT, UDP flavonoid gulcosyl transferase; OMT, O-methyltransferase; PAL, phenylalanine ammonia lyase; RT, rhamnosyl transferase. The products of each enzymatic reaction are listed below the arrows. Colored circles indicate the presence of delphinidin and cyanidin anthocyanidins, X represents absence of pelargonidin.

Accumulation of the secondary metabolite anthocyanin is predominantly responsible for red, blue, and purple pigmentation in angiosperms. Pigmentation is a major determinant of a species’ pollination syndrome, which refers to the selection of particular floral traits caused by the preference of their pollinators (Fenster et al., 2004). Flower color is intricately regulated by the specific combinations of certain pigment metabolites produced, and is subjected to ecological selection and convergent evolution. Therefore, flower color is an ideal trait for examining ecological and evolutionary selection processes. The anthocyanin biosynthetic pathway (ABP) has been well established in many model species, such as *Arabidopsis*, petunia (*Petunia hybrida* E. Vilm.), and snapdragon (*Antirrhinum majus* L.) (Buer et al., 2010; Pollastri & Tattini, 2011). Most of the knowledge of anthocyanin biosynthesis in *Arabidopsis* has been obtained from the analysis of transparent testa (*tt*) mutants, which show loss of seed pigmentation (Lepiniec et al., 2006). In the early steps of the pathway, the key enzymes chalcone synthase (CHS), chalcone isomerase (CHI), and flavanone 3-hydroxylase (F3H) condense and convert a phenylpropanoid precursor, *p*-coumaroyl-CoA, along with three molecules of malonyl CoA, to dihydrokaempferol (Lepiniec et al., 2006). Parallel catalyzation by flavonoid-3’-hydroxylase, flavonoid-3’,5’-hydroxylase, dihydroflavonol-4-reductase (DFR), and anthocyanidin synthase (ANS) results in the production of various types of anthocyanidin (**Figure 1**) (Holton & Cornish, 1995; Boss et al., 1996). The transcriptional regulators controlling flavonoid biosynthetic enzymes have been extensively studied, and include the MYB, the bHLH, and the WD-repeat proteins. Yeast-three-hybrid protein interaction data suggested that a protein complex of the MYB-bHLH-WD40 transcription factors binds the regulatory promoter regions of the flavonoid pathway enzymatic, or structural, genes, to regulate anthocyanin biosynthesis (Gonzalez et al., 2008).

The evolutionary basis for the loss of anthocyanin pigments in floral tissue has been investigated by characterizing major floral pigmentation loci using controlled cross segregating populations (Schwinn, 2006; Whittall et al., 2006; Hoballah et al., 2007; Streisfeld and Rausher, 2009; Smith and Rausher, 2011). Evidence suggests that flower color transition is affected by the transcriptional regulation of several anthocyanin structural genes expression. For example, altered activity of specific transcriptional factors accounts for altered patterns of pigmentation in white *Petunia axillaris* and some *Antirrhinum* species (Schwinn, 2006; Hoballah et al., 2007). *Cis*-regulatory changes in the *F3’H* gene promoter cause down-regulation of *F3’H* transcription and altered flux in the anthocyanin pathway, resulting in increased production of the red pigment, pelargonidin, instead of blue, in *Ipomoea horsfalliae* Hook. (Des Marais & Rausher, 2010). Although it has been suggested that mutations in structural genes may incur higher deleterious pleiotropy than those in *cis*-regulatory elements or transcription factors, the possibility that enzyme coding sequence variation is involved in flower color transition cannot be excluded (Streisfeld and Rausher, 2009).

**Figure 2:**
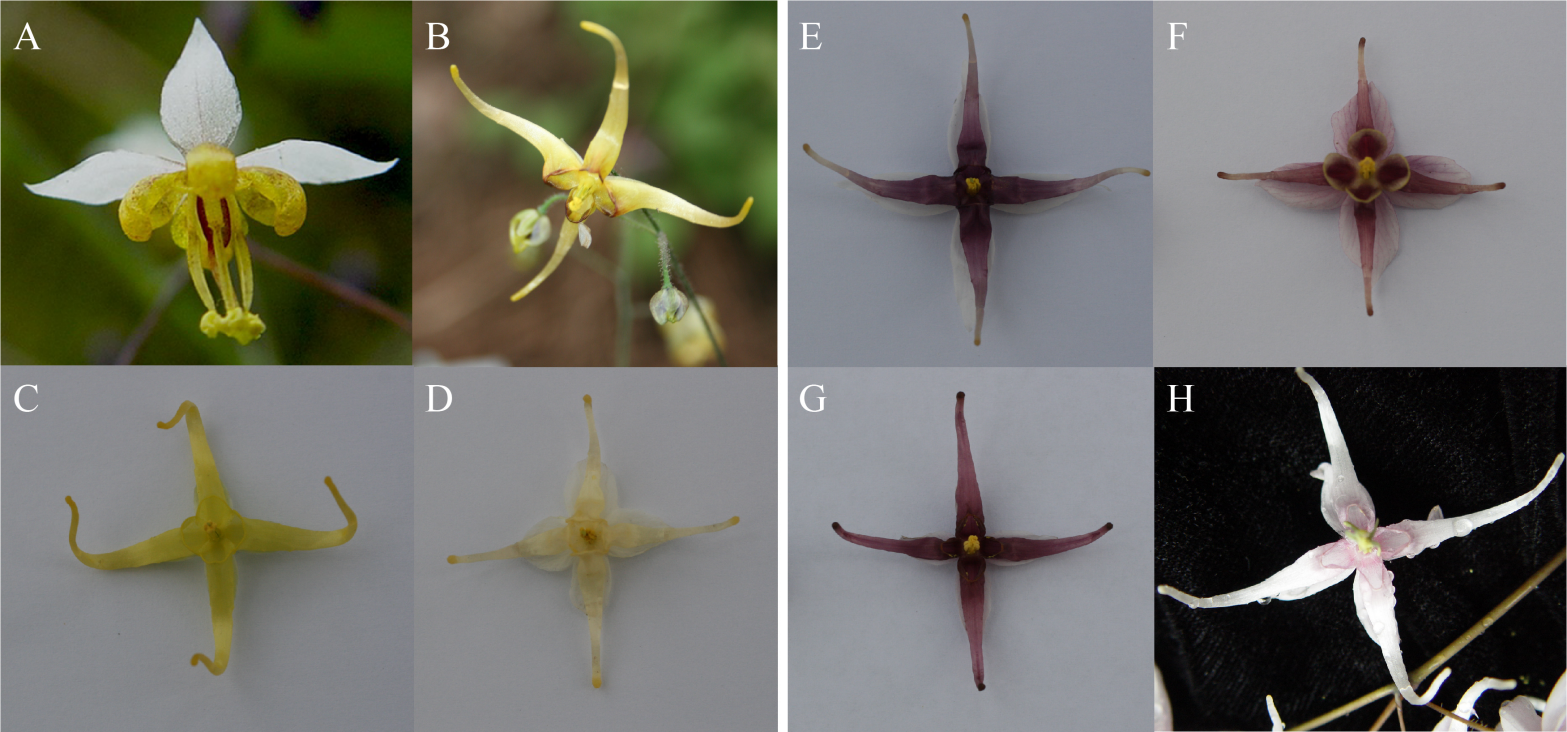
Floral phenotypes of accessions of different species within the genus *Epimedium*. **(A–D)** are non-pigmentation species (A-); **(E–H)** are classified as pigmentation species (A+). All photos were taken by W. S. **(A)** *E. sagittatum*, **(B)** *E. lishihchenii*, **(C)** *E. franchetii*, **(D)** *E. wushanense*, **(E)** *E. zhushanense*, **(F)** *E. epstenii*, **(G)** *E. acuminatum*, **(H)** *E. leptorrhizum*.

The *Epimedium* genus (Berberidaceae), known as “Yinyang Huo” by Chinese druggists, is one of the most popular traditional Chinese medicinal herb genera (Sun et al., 2014; Zhang et al., 2021). A monophyletic group of 50 species of *Epimedium* is found in western and central China (Huang et al., 2013a; Huang et al., 2013b; Huang et al., 2015; Huang et al., 2016). *Epimedium* species display a vast range of flower colors; from white and yellow to rose, crimson, and violet (**Figure 2**). These color pigments are distributed in petaloid sepals or petals or both. In this study, we studied the phenotypic variation of color in *Epimedium* species distributed in the Hubei province of China. The expression of genes involved in the anthocyanin biosynthetic pathway (ABP) was also analyzed for the association with the different flower color polymorphisms. Our study focused on answering two questions: (1) Has anthocyanin pigment loss, or variation, in different species resulted from the same mechanism? (2) Which candidate genes are involved in anthocyanin pigmentation in *E. sagittatum*?

## Results

### Analysis of pigments and flavonoid intermediates in different *Epimedium* species

**Figure 3:**
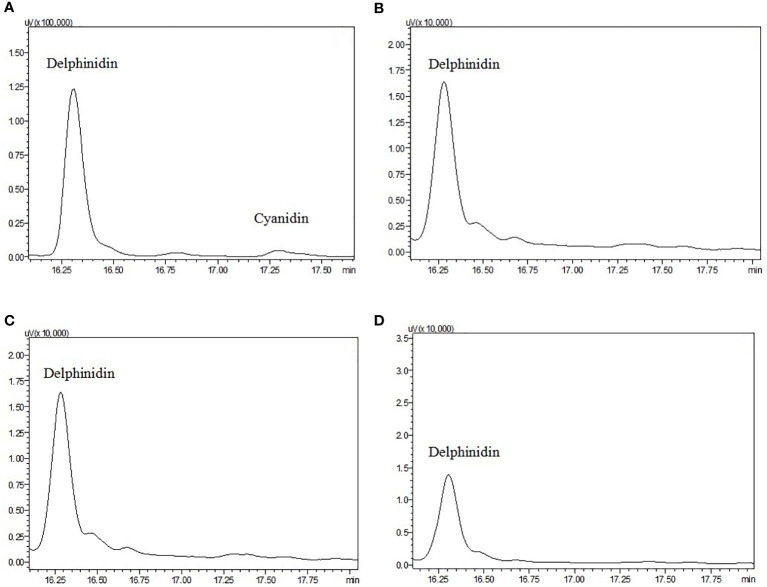
High-performance liquid chromatograms of extracts from petals and sepals of anthocyanidin pigments from **(A)** *E. acuminatum*, **(B)** *E. epstenii*, **(C)** *E. zhushanense* and **(D)** *E. leptorrhizum*. Peaks labeled represent the standards of each of the anthocyanin.

Using HPLC, the major pigments from the floral tissues of anthocyanin species (A+) species were found to comprise delphinidin and cyanidin, whereas no detectable anthocyanins were found in the non-anthocyanin species (A-) flowers (**Figure 3**). To further characterize the mechanism responsible for the non-pigmentation of flowers in A- species, *E. sagittatum* was used as a model for the enzymatic function.

### Expression of ABP genes in floral tissues of *Epimedium*

**Figure 4:**
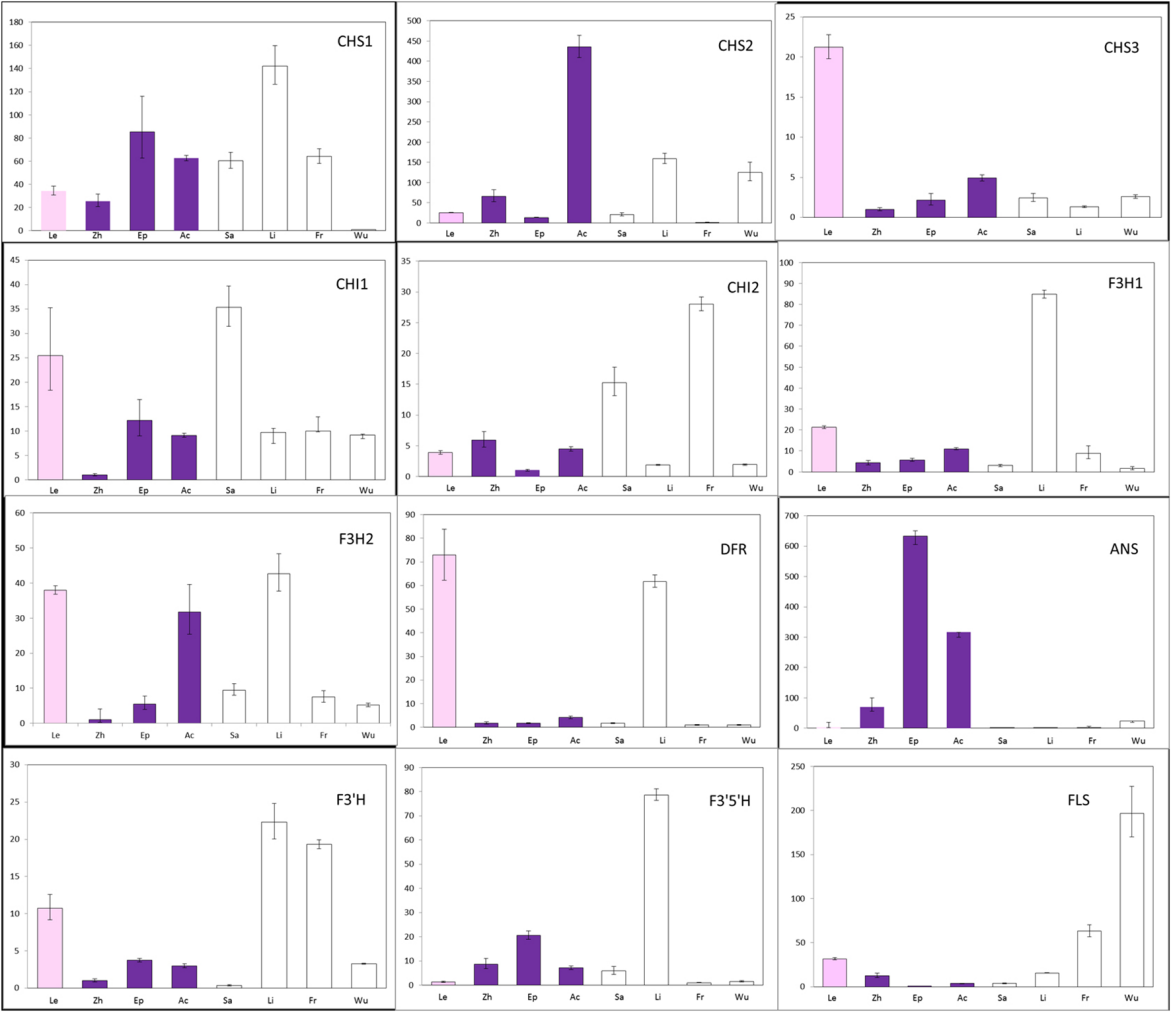
Quantitative expression pattern of ABP structural genes from petal tissue of eight *Epimedium* species. Colored and empty bars represent A+ and A- species, respectively. Le, Zh, Ep, Ac, Sa, Li, Fr and Wu represent *E. leptorrhizum*, *E. zhushanense, E. epstenii*, *E.acuminatum*, *E. sagittatum*, *E. lishihchenii, E. franchetii* and *E. wushanense*. Data presented here are the mean values of three replicates with error bars indicating SE.

To determine whether changes in gene expression might be involved in the non-pigmentation phenotype of A- species, the transcript levels of putative anthocyanin biosynthetic enzymes were examined in petal tissue (**Figure 4**; **Supplementary Figure 1A**). Expression of *CHS1* not *CHS2* and *CHS3* was found to be significantly lower in *E. wushanese* (A-) than in other species. Similarly, down-regulation of *CHS2* was observed in *E. frachetii* (A-), suggesting that loss of anthocyanin may result from low levels of expression of different copies of *CHS* in *E. wushanese* and *E. franchetii*. For *CHI* and *F3H*, we found no significant correlation between expression level and the loss of anthocyanins in spur tissues of all A- species. Among the structural genes, *ANS* was the only ABP locus where all A- species had significantly lower expression levels than that of A+ species. This suggests that the lack of pigmentation production in all A- species could be caused primarily by lower *ANS* expression. The expression level of *DFR* was significantly lower in A- species than in A+ species, except for *E. lishihchenii*. It has been reported that substrate competition between *FLS* and *DFR* creates a metabolic flux of the flavonoid biosynthetic pathway in *Arabidopsis*. In this study, low expression of *DFR* in the A- species *E. franchetii* and *E. wushanese* was correlated with increased accumulation of *FLS* expression. On the other hand, up-regulation of *DFR* was positively correlated with *FLS* expression in *E. lishihchenii* but *E. sagittatum* showed no correlation with *DFR*. In summary, these results suggest that the loss of anthocyanin in *E. frachetii*, *E. wushanese* and *E. sagittatum* may be primarily related to alterations at the *ANS* locus, affecting gene expression.

**Figure 5:**
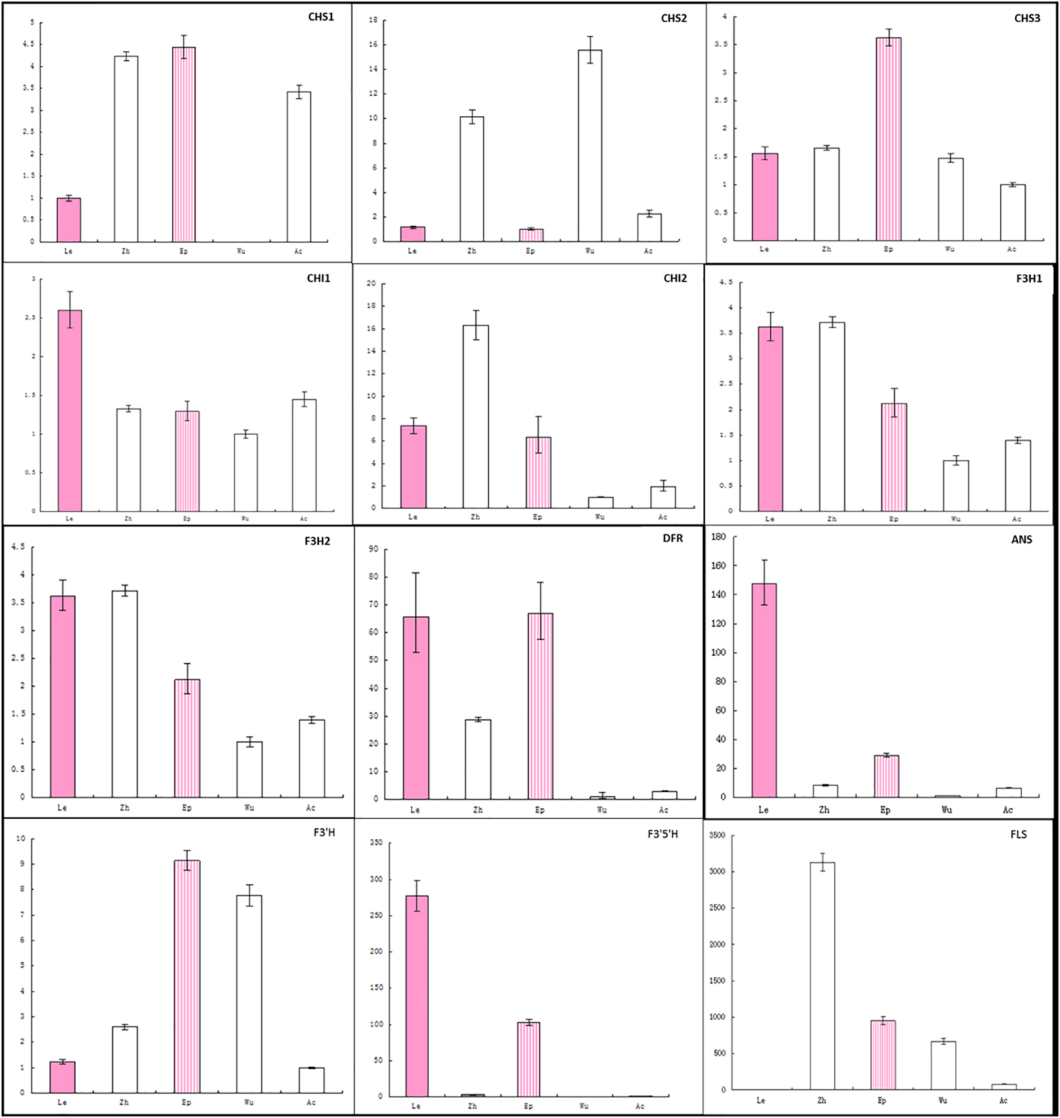
Analysis of expression profiles of anthocyanin genes in petaloid sepals of five *Epimedium* species using real-time PCR. The cDNA templates are listed as follows: Le, *E. leptorrhizum*; *Zh*, *E. zhushanense*; *Ep*, *E. epstenii*; Wu, *E. wushanense* and Ac, *E. acuminatum*. Data presented here are the mean values of three replicates with error bars indicating SE.

To further analyze the loss of anthocyanin in sepals (**Figure 5**; **Supplementary Figure 1B**), gene expression was analyzed across five *Epimedium* species using the same primers. Expression of *DFR* was lowest in the three A- species and was correlated with *ANS* expression, suggesting the expression of *DFR* and *ANS* could be regulated by a common transcription factor. *CHS1* transcripts were not detected in the sepals of *E. wushanese*, which also had the lowest *CHS1* expression in petals. These observations suggest that negative regulation of the *DFR* and *ANS* genes together was also correlated with the lowest *CHS1* in sepals and petals in *E. wushanese*.

### Complementation analyses

**Figure 6:**
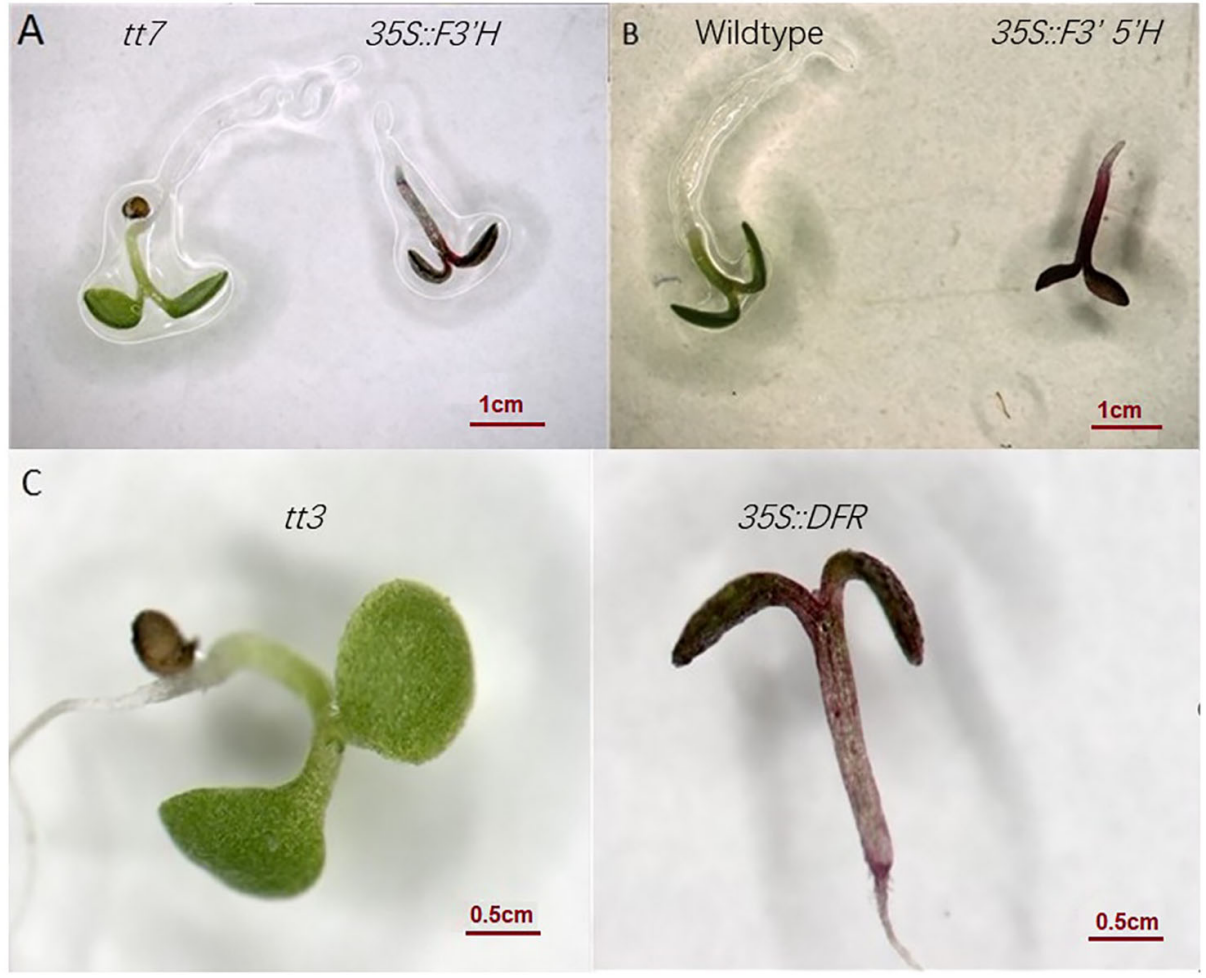
Phenotypes of the *Arabidopsis* with overexpression of *EsF3’H*, *EsDFR and F3’5’H***(A)** Image of anthocyanin in *tt7* mutant and rescuing line of *EsF3’H* in *tt7* background, **(B)** Phenotypes of wild-type, and transgenic *Arabidopsis* seedling with *EsF3’5’H*, **(C)** Phenotypes of *tt3* mutant, and transgenic *Arabidopsis* seedling with *EsDFR*.

To study the catalytic activity of *E. sagittatum* ABP gene products, 35S::*EsF3’H* and 35S::*EsDFR* genes were individually transferred into their respective *Arabidopsis* mutants; *transparent testa 7* (*tt7)* lacking flavonoid 3’-hydroxylase, and *transparent testa3* (*tt3*) lacking dihydroflavonol reductase under the control of the cauliflower mosaic virus 35S promoter (Peer et al., 2001). Transgenic and mutant control seedlings were grown under nitrogen stress to determine if the *Epimedium* genes could rescue the *Arabidopsis* anthocyanin-null mutant phenotypes. Accumulation of anthocyanins was observed in transgenic seedlings ectopically expressing *EsF3’H* and *EsDFR* (**Figure 6**). However, the *tt7* and *tt3* mutant controls did not exhibit anthocyanin accumulation in cotyledons. Thus the *E. sagittatum* genes showed catalytic activity in *Arabidopsis*. Given the lack of an *Arabidopsis* mutant for *F3’5’H*, in order to determine whether *EsF3’5’H* can function *in vivo*, we overexpressed *35S::EsF3’5’H* in wild-type *Arabidopsis*. Under normal conditions on 1/2 MS medium, the seedlings overexpressing *EsF3’5’H* showed comparable anthocyanin production to wild-type controls (**Figure 6**).

## Discussion

The four *Epimedium* A- species (*E. sagittatum*, *E. lishihchenii*, *E. franchetii* and *E. wushanense*) investigated in this study appeared to exhibit anthocyanin loss at the phenotypic level *via* reduced activity of the anthocyanin branch of the flavonoid pathway. In all species, this appears to involve reduced transcriptional activity of pathway genes, similar to studies in *Mimulus aurantiacus* (Streisfeld & Rausher, 2009). Interestingly, one A- species (*E. lishihchenii*) expressed all ABP loci except for *ANS* at a high level.

While the data linking conserved gene regulation changes to anthocyanin level changes are purely correlative, we found no evidence for the role of coding-region mutations in determining different anthocyanin levels. In A- specie *E. sagittatum*, the F3’H and DFR enzymes were shown to rescue anthocyanin production in their corresponding *Arabidopsis* mutants, suggestive of adequate catalytic function (Huang et al., 2012). Accumulation of anthocyanin in *35S::EsDFR* in this study and *35S::EsMYBA1* transformed *Arabidopsis* indicated functionality of the *EsDFR* and *EsMYBA1* coding region (Huang et al., 2013a). Thus, we concluded that the loss of flower color in *E. sagittatum* (A-) was due to a tissue-specific regulatory change affecting *EsDFR* and *EsANS* transcription and not coding-region mutations of *EsDFR*, *EsANS*, or *EsMYBA1*. We also suggested that the changes responsible for the loss of pigmentation in *E. franchetii* and *E. wushanense* flowers were shared with *E. sagittatum*, based on similar correlative gene expression patterns and anthocyanin production in leaves. Functional assays of the putative *cis*-elements and trans-regulators involved in *DFR* or *ANS* transcription were required to determine the precise regulatory mechanisms resulting in reduced *ANS* and *DFR* gene expression in A- species.

Downregulation of *CHS* was a major cause of white flowers in natural populations of *Aquilegia flavellata* and *Parrya nudicaulis* (Whittall et al., 2006; Dick et al., 2011). Although we found an association between the A- phenotype and downregulation of *CHS1* in yellow-flowered *E. wushanese*, *DFR*, and *ANS* were also downregulated, which may also have contributed to the A- phenotype. Therefore, the A- phenotype in four *Epimedium* species was also proposed to be due to alteration at the regulatory level, rather than functional mutations in ABP enzymes. The loci regulating anthocyanin in *Epimedium* were currently being fine-mapped and confirmed by transformation assays.

## Materials and methods

### Tissue harvest

Eight *Epimedium* species (*E. acuminatum*, *E. franchetii*, *E. leptorrhizum*, *E. epstenii*, *E. sagittatum*, *E. lishihchenii*, *E. wushanense*, *and E. zhushanense*) grown in the specialized *Epimedium* nurseries of Wuhan Botanical Garden, Chinese Academy of Sciences, Wuhan, China (**Figure 2**). All plants were transplanted from wild populations and growing under the same environmental conditions. Floral tissues including petaloid sepals and spur-like petals were collected in the spring of 2011. The eight species were separated into two groups corresponding to anthocyanin (A+) (*E. acuminatum*, *E. leptorrhizum, E. epstenii* and *E. zhushanense*) and non-anthocyanin (A-) (*E. franchetii*, *E. sagittatum*, *E. lishihchenii*, *E. wushanense*) based on visual observation of the floral tissues. The samples were weighed, packaged in aluminum foil, flash-frozen in liquid nitrogen, and then stored at -80°C.

### HPLC analysis of flavonoid intermediates and anthocyanin

The profiles of anthocyanidins from the samples of A- species and A+ species were analyzed using HPLC. The precursors of anthocyanin pigments were extracted from 100 mg of fresh corolla tissue. For each sample, 20 μL of supernatant was injected into a Shimadzu LC-20 AT liquid chromatograph (Shimadzu Corporation, Japan) and a 250×4.6 mm reverse phase C18 column (Sigma-Aldrich, USA) at a flow rate of 1 ml min^-1^. The organic solvent was composed of acetonitrile and 0.1% trifluoroacetic acid, and the polar solvent was 0.1% trifluoroacetic acid in HPLC-grade water. The anthocyanin was measured at 550 nm. The chemical compounds cyanidin, delphinidin, malvidin, pelargonidin, peonidin, and petunidin (Poypehenols Laboratories, Norway), were used as anthocyanidin standards.

### Transferring ABP candidate genes into other *Epimedium* species

In total, 12 genes from *E. sagittatum* involved in the ABP were cloned following RT-PCR amplification using degenerate primers or specific primers based on our previous investigation(Zeng et al., 2010; Huang et al., 2013a; Huang et al., 2013b; Huang et al., 2015). These genes were *CHS1*, *CHS2*, *CHS3*, *CHI1*, *CHI2*, *F3H1*, *F3H2*, *F3’H*, *F3’5’H*, *FLS*, *DFR*, *ANS*. In this study, all pairs of primer from *E. sagittatum* were transferred to other *Epimedium* species.

### Gene expression

Total RNA was extracted from inner sepals and petals at anthesis, at which time the biosynthesis of anthocyanin is completed. First-strand cDNA was synthesized using PrimeScript RT reagent Kit (Takara, Japan) following the manufacturer’s instructions. In each 20 μL qRT-PCR reaction, 50 ng of cDNA was amplified using SYBR^®^ Premix Ex TaqTM II (Takara, Japan) and 100 mM of primers in an ABI7500 Real-Time PCR machine (ABI, USA) as per the manual. Actin was amplified as the control gene. The samples from three tissues were used and three technical replicates were performed for each sample. Data were analyzed by ABI7500 software. In this study, all pairs of primer (*CHS1*, *CHS2*, *CHS3*, *CHI1*, *CHI2*, *F3H1*, *F3H2*, *F3’H*, *F3’5’H*, *FLS*, *DFR*, and *ANS*) from *E. sagittatum* were transferred in other *Epimedium* species. All primers used in this manuscript are listed in the supplementary database.

### Complementation analysis

For functional analyses, the *E. sagittatum* (A-) genes *EsF3’H* and *EsDFR* were overexpressed in their respective *Arabidopsis thaliana* (ecotype Landsberg) mutants, each lacking anthocyanins at the seedling stage. *EsF3’5’H*, The coding regions of *EsF3’H*, *EsF3’5’H*, and *EsDFR* were cloned into pMD19-T (Takara, Japan). The SalI and SacI digested fragment of each gene was purified and ligated into the pMV plasmid (derived from pBI121) behind the cauliflower 35S promoter. The plasmids were then transformed into *Agrobacterium* strain EHA105. *Arabidopsis* wild-type and mutants (*tt3* and *tt7*) were transformed by the floral dip infiltration method(Zhang et al., 2006). Transformants were selected on 1/2 Murashige and Skoog medium supplemented with 50 μg/mL kanamycin. Resistant seedlings were then transferred into the soil to harvest seeds. T1 seedlings were screened on 1/2 MS medium minus nitrogen for observation of anthocyanin accumulation.

## Data availability statement

Publicly available datasets were analyzed in this study. This data can be found here: Reference.

## Author contributions

WS, HH, and YW conceived and designed the experiments. WS and YM performed the experiments.WS, WH, ZY, XM, and HW analyzed the data. WS wrote the paper. XM and HW revised the paper. YZ provided **Figure 2** and collected species. RH and SC supervised this investigation. All authors contributed to the article and approved the submitted version.

## References

[B1] Boss P. K. Davies C. Robinson S. P. (1996). Analysis of the expression of anthocyanin pathway genes. Plant Physiol. 111, 1059–1066. doi: 10.1104/pp.111.4.1059 12226348 PMC160981

[B2] Buer C. S. Imin N. Djordjevic M. A. (2010). Flavonoids: New roles for old molecules. J. Integr. Plant Biol. 52, 98–111. doi: 10.1111/j.1744-7909.2010.00905.x 20074144

[B3] Des Marais D. L. Rausher M. D. (2010). Parallel evolution at multiple levels in the origin of hummingbird pollinated flowers in *Ipomoea* . Evol. (N. Y). 64, 2044–2054. doi: 10.1111/j.1558-5646.2010.00972.x 20148948

[B4] Dick C. A. Buenrostro J. Butler T. Carlson M. L. Kliebenstein D. J. Whittall J. B. (2011). Arctic Mustard flower color polymorphism controlled by petal-specific downregulation at the threshold of the anthocyanin biosynthetic pathway. PLoS One 6. doi: 10.1371/journal.pone.0018230 PMC3072389 21490971

[B5] Fenster C. B. Armbruster W. S. Wilson P. Dudash M. R. Thomson J. D. (2004). Pollination syndromes and floral specialization. Annu. Rev. Ecol. Evol. Syst. 35, 375–403. doi: 10.1146/annurev.ecolsys.34.011802.132347

[B6] Gonzalez A. Zhao M. Leavitt J. M. Lloyd A. M. (2008). Regulation of the anthocyanin biosynthetic pathway by the TTG1/bHLH/Myb transcriptional complex in *Arabidopsis* seedlings. Plant J. 53, 814–827. doi: 10.1111/j.1365-313X.2007.03373.x 18036197

[B7] Hoballah M. E. Gubitz T. Stuurman J. Broger L. Barone M. Mandel T.. (2007). Single gene-mediated shift in pollinator attraction in *Petunia* . Plant Cell 19, 779–790. doi: 10.1105/tpc.106.048694 17337627 PMC1867374

[B8] Holton T. A. Cornish E. C. (1995). Genetics and biochemistry of anthocyanin biosynthesis. Plant Cell 7, 1071. doi: 10.2307/3870058 12242398 PMC160913

[B9] Huang W. Khaldun A. B. M. Lv H. Du L. Zhang C. Wang Y. (2016). Isolation and functional characterization of a R2R3-MYB regulator of the anthocyanin biosynthetic pathway from *Epimedium sagittatum* . Plant Cell Rep. 35, 883–894. doi: 10.1007/s00299-015-1929-z 26849670

[B10] Huang W. Sun W. Lv H. Luo M. Zeng S. Pattanaik S.. (2013a). A R2R3-MYB transcription factor from *Epimedium sagittatum* regulates the flavonoid biosynthetic pathway. PLoS One 8. doi: 10.1371/journal.pone.0070778 PMC3731294 23936468

[B11] Huang W. Sun W. Lv H. Xiao G. Zeng S. Wang Y. (2013b). Isolation and molecular characterization of thirteen R2R3-MYB transcription factors from *Epimedium sagittatum* . Int. J. Mol. Sci. 14, 594–610. doi: 10.3390/ijms14010594 PMC3565284 23271373

[B12] Huang W. Sun W. Wang Y. (2012). Isolation and molecular characterisation of flavonoid 3’-hydroxylase and flavonoid 3’, 5’-hydroxylase genes from a traditional Chinese medicinal plant, *Epimedium sagittatum* . Gene 497, 125–130. doi: 10.1016/j.gene.2011.11.029 22248626

[B13] Huang W. Zeng S. Xiao G. Wei G. Liao S. Chen J.. (2015). Elucidating the biosynthetic and regulatory mechanisms of flavonoid-derived bioactive components in *Epimedium sagittatum* . Front. Plant Sci. 6. doi: 10.3389/fpls.2015.00689 PMC4558469 26388888

[B14] Lepiniec L. Debeaujon I. Routaboul J.-M. Baudry A. Pourcel L. Nesi N.. (2006). Genetics and biochemistry of deed flavonoids. Annu. Rev. Plant Biol. 57, 405–430. doi: 10.1146/annurev.arplant.57.032905.105252 16669768

[B15] Peer W. A. Brown D. E. Tague B. W. Muday G. K. Taiz L. Murphy A. S. (2001). Flavonoid accumulation patterns of transparent testa mutants of *Arabidopsis* . Plant Physiol. 126, 536–548. doi: 10.1104/pp.126.2.536 11402185 PMC111147

[B16] Pollastri S. Tattini M. (2011). Flavonols: Old compounds for old roles. Ann. Bot. 108, 1225–1233. doi: 10.1093/aob/mcr234 21880658 PMC3197460

[B17] Schwinn K. (2006). A small family of MYB-regulatory genes controls floral pigmentation intensity and patterning in the genus *Antirrhinum* . Plant Cell 18, 831–851. doi: 10.1105/tpc.105.039255 16531495 PMC1425845

[B18] Smith S. D. Rausher M. D. (2011). Gene loss and parallel evolution contribute to species difference in flower color. Mol. Biol. Evol. 28, 2799–2810. doi: 10.1093/molbev/msr109 21551271 PMC3203625

[B19] Streisfeld M. A. Rausher M. D. (2009). Genetic changes contributing to the parallel evolution of red floral pigmentation among *Ipomoea* species. New Phytol. 183, 751–763. doi: 10.1111/j.1469-8137.2009.02929.x 19594698

[B20] Sun W. Huang W. Li Z. Song C. Liu D. Liu Y.. (2014). Functional and evolutionary analysis of the AP1/SEP/AGL6 superclade of MADS-box genes in the basal eudicot *Epimedium sagittatum* . Ann. Bot. 113, 653–668. doi: 10.1093/aob/mct301 24532606 PMC3936592

[B21] Whittall J. B. Voelckel C. Kliebenstein D. J. Hodges S. A. (2006). Convergence, constraint and the role of gene expression during adaptive radiation: Floral anthocyanins in *Aquilegia* . Mol. Ecol. 15, 4645–4657. doi: 10.1111/j.1365-294X.2006.03114.x 17107490

[B22] Zeng S. Xiao G. Guo J. Fei Z. Xu Y. Roe B. A.. (2010). Development of a EST dataset and characterization of EST-SSRs in a traditional Chinese medicinal plant, *Epimedium sagittatum* (Sieb. et zucc.) maxim. BMC Genomics 11. doi: 10.1186/1471-2164-11-94 PMC2829513 20141623

[B23] Zhang X. Henriques R. Lin S. S. Niu Q. W. Chua N. H. (2006). Agrobacterium-mediated transformation of *Arabidopsis thaliana* using the floral dip method. Nat. Protoc. 1, 641–646. doi: 10.1038/nprot.2006.97 17406292

[B24] Zhang Y. Li J. Wang Y. Liang Q. (2021). Taxonomy of Epimedium (Berberidaceae) with special reference to Chinese species. Chin. Herb. Med. 14 (1), 20–35. doi: 10.1016/j.chmed.2021.12.001 36120133 PMC9476710

