# Supplementary figures and images for: Genetic and molecular analysis of the anthocyanin pigmentation pathway in Epimedium
Source: Front Plant Sci. 2023 Mar 27;14:1133616. doi: 10.3389/fpls.2023.1133616 (PMC10090855; doi:10.3389/fpls.2023.1133616)

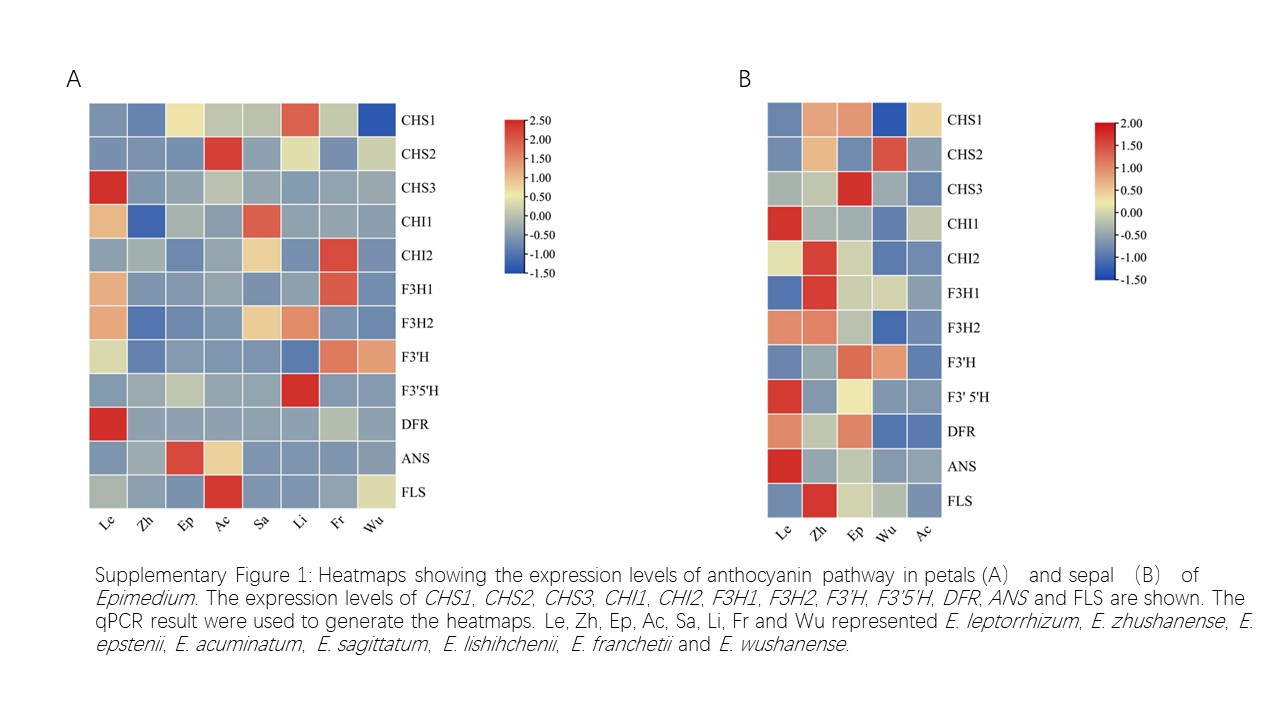

Supplement: Supplementary file 1 [file Image_1.jpeg]
